# Marine biorhythms: bridging chronobiology and ecology

**DOI:** 10.1098/rstb.2016.0253

**Published:** 2017-10-09

**Authors:** Martin Bulla, Thomas Oudman, Allert I. Bijleveld, Theunis Piersma, Charalambos P. Kyriacou

**Affiliations:** 1 NIOZ Royal Netherlands Institute for Sea Research, Department of Coastal Systems, Utrecht University, PO Box 59, 1790 AB Den Burg, The Netherlands; 2 Department of Ecology, Faculty of Environmental Sciences, Czech University of Life Sciences Prague, Kamýcká 129, 165 21 Prague 6, Suchdol, Czech Republic; 3 Department of Behavioural Ecology and Evolutionary Genetics, Max Planck Institute for Ornithology, Eberhard Gwinner Str., 82319 Seewiesen, Germany; 4 Conservation Ecology Group, Groningen Institute for Evolutionary Life Sciences (GELIFES), University of Groningen, PO Box 11103, 9700 CC Groningen, The Netherlands; 5 Department of Genetics, University of Leicester, Leicester LE1 7RH, UK

**Keywords:** circadian, tidal, lunar, shorebirds, invertebrates, molecular

## Abstract

Marine organisms adapt to complex temporal environments that include daily, tidal, semi-lunar, lunar and seasonal cycles. However, our understanding of marine biological rhythms and their underlying molecular basis is mainly confined to a few model organisms in rather simplistic laboratory settings. Here, we use new empirical data and recent examples of marine biorhythms to highlight how field ecologists and laboratory chronobiologists can complement each other's efforts. First, with continuous tracking of intertidal shorebirds in the field, we reveal individual differences in tidal and circadian foraging rhythms. Second, we demonstrate that shorebird species that spend 8–10 months in tidal environments rarely maintain such tidal or circadian rhythms during breeding, likely because of other, more pertinent, temporally structured, local ecological pressures such as predation or social environment. Finally, we use examples of initial findings from invertebrates (arthropods and polychaete worms) that are being developed as model species to study the molecular bases of lunar-related rhythms. These examples indicate that canonical circadian clock genes (i.e. the homologous clock genes identified in many higher organisms) may not be involved in lunar/tidal phenotypes. Together, our results and the examples we describe emphasize that linking field and laboratory studies is likely to generate a better ecological appreciation of lunar-related rhythms in the wild.

This article is part of the themed issue ‘Wild clocks: integrating chronobiology and ecology to understand timekeeping in free-living animals’.

## 1. Introduction

**Figure 1.**
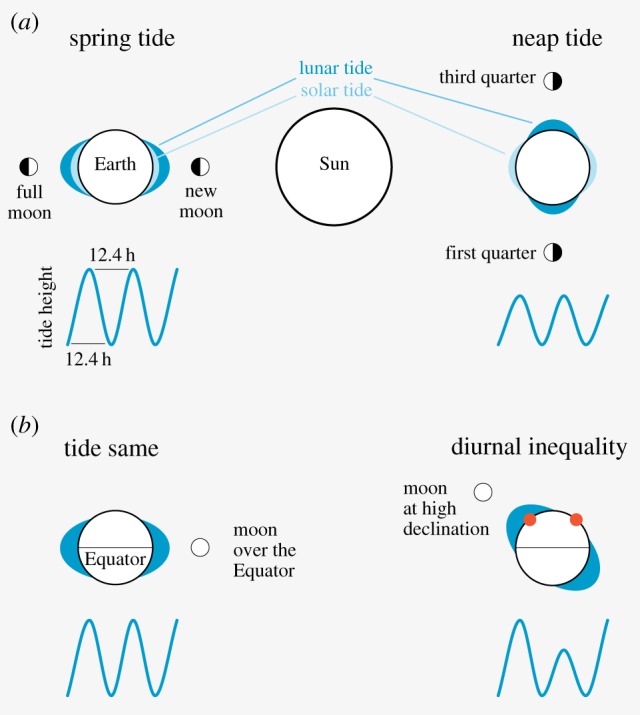
Variation in high-tide levels. (*a*) When the Sun, Moon and Earth are in alignment during new or full moon (i.e. twice a month) the gravitational pull on the oceans is strongest, producing the high-amplitude spring tides, i.e. lunar tide (dark blue) and sun tide (light blue) combine. In contrast, when the Moon is in its first or third quarter the gravitational pull on the oceans is reduced, leading to the low amplitude neap tides. (*b*) If the Moon orbits directly over the Equator, the day and night tides are similar, whereas when the Moon orbits at high declination the night tides are higher than the day tides (diurnal inequality; indicated by red dots).

As the Earth rotates around its axis every 24 h, it generates relentless rhythms of light and dark, heat and cold. In addition, the tilt of the Earth's axis produces the annual seasonal rhythms that so dramatically modulate the light and dark cycles as we move towards the polar extremes [1,2]. The rotation of the Earth and the gravitational pull of the Sun and the Moon deform the mass of the oceans, producing the rise and fall of sea levels every 12.4 h. When the Earth, Moon and Sun are in alignment during new and full moon every 15 days, the gravitational pull on the Earth's oceans is at its maximum, producing the high-amplitude spring tides (figure 1*a*). When the Sun and Moon are at right angles when viewed from the Earth (Moon's first or third quarter), the gravitational pull on the oceans is reduced, generating the low-amplitude neap tides (figure 1*a*). Furthermore, when the Moon orbits off the equatorial plane the tide is higher at night than during the day, a phenomenon termed ‘diurnal inequality’ ([3] and figure 1*b*). Finally, there is the waxing and waning of the Moon itself with its 14.8 day semi-lunar and 29.6 day lunar cycles.

For hundreds of millions of years these geophysical cycles have shaped the behaviour and physiology of organisms. Not surprisingly, nearly all terrestrial and marine species (including some bacteria) show circadian phenotypes [4]. In addition, organisms living in intertidal zones also show tidal, semi-lunar and lunar cycles [5]. However, marine biorhythms are rarely studied in higher vertebrates [6]. Also, whereas genetic studies of circadian rhythms have a 45-year history, particularly in the model organisms of mouse and *Drosophila*, until recently a similar approach to studying rhythms in intertidal (non-model) organisms was not feasible. However, in the past few years, the advent of genomic technologies that are applicable to any species has initiated the mechanistic study of tidal and lunar cycles of behaviour and physiology [7].

Here, our aims are threefold. We first address the scarcity of data on intertidal higher vertebrates by investigating the interactions between tidal and daily cycles in the foraging movements and incubation rhythms of shorebirds. We then discuss some fresh studies that have illuminated the role of circadian clock genes in the intertidal behaviour and physiology of arthropods and worms. Finally, we use our findings and the reported examples to highlight how collaborations between field ecologists and chronobiologists may uncover fundamental adaptive principles about biorhythms in the wild.

## 2. Tidal rhythms in shorebirds

Substantial numbers of shorebird species live and feed, at least for part of the year, in tidal habitats [8,9]. Some of these tidal populations are sedentary in tidal environments, and face day–night fluctuations of illumination throughout the year (e.g. several species of oystercatcher, *Haematopus*; [10]). Other populations are migratory and live in the coastal nonbreeding areas during 8–10 months of the year, where they cope with a combination of tidal and day–night environmental rhythms (e.g. bar-tailed godwit, *Limosa lapponica*; sanderling, *Calidris alba*; and red knot, *Calidris canutus*), and breed in Arctic non-tidal environments for two months of the year, where day–night environmental rhythms are damped [8,9]. Shorebirds manage the interplay between circadian and tidal environmental, but how they schedule their behaviour to the interacting environmental rhythms is unclear [11]. Indeed, the behavioural rhythms of shorebirds under such circumstances are relatively unexplored (but see [12,13]).

To anticipate tidal foraging opportunities, it is assumed that these species have activity patterns with a period length resembling the tidal period. We might expect shorebirds that use tides throughout the whole year to exhibit incubation rhythms with tidal periods [14] more readily than shorebirds that only use tides away from their breeding grounds. Nevertheless, as changing to a different rhythm may be costly [15], the tidal activity patterns could carry over to incubation even for shorebirds that are tidal only when away from their breeding grounds.

The aims of our shorebird study are twofold. We used novel automated-tracking technology [16] to first describe the foraging rhythms of red knots at Banc d'Arguin, their coastal Mauritanian wintering ground—an environment with both tidal rhythms and strong diel fluctuations in light intensity (see [17]. Second, we analyse data from a recent comparative study on shorebirds that incubate biparentally [14,18], to reveal whether shorebirds with tidal life-histories keep tidal rhythms also during incubation [14].

### (a) The tidal rhythm of red knots

Red knots, *C. canutus*, are long-distance migratory shorebirds that breed in the High Arctic and live in coastal intertidal environments during the rest of the year [19,20], where they almost exclusively eat hard-shelled molluscs ingested whole and crushed in their large muscular gizzards [21]. When the tide goes out and the intertidal mudflats become available they take the opportunity to feed, being forced to retreat to shoreline high-tide roost during the high-water periods [22]. However, the individual variation in foraging rhythm of knots (and of any other intertidal bird) is unknown.

**Figure 2.**
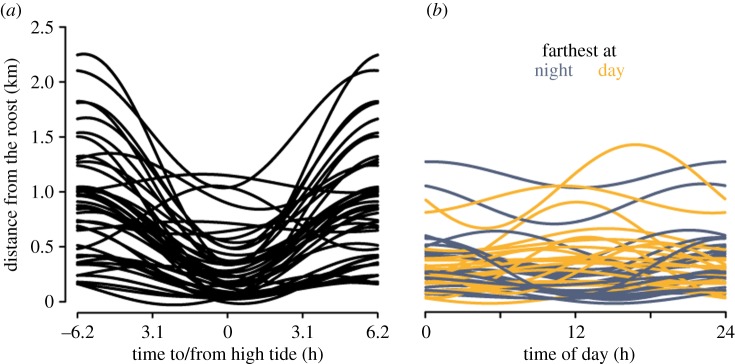
Distance of redknots to their to the closest roost relative to high tide (*a*) and time of day (*b*). Each line depicts the model prediction for a single individual (*N* = 42 individuals; see [16] for details.

**Figure 3.**
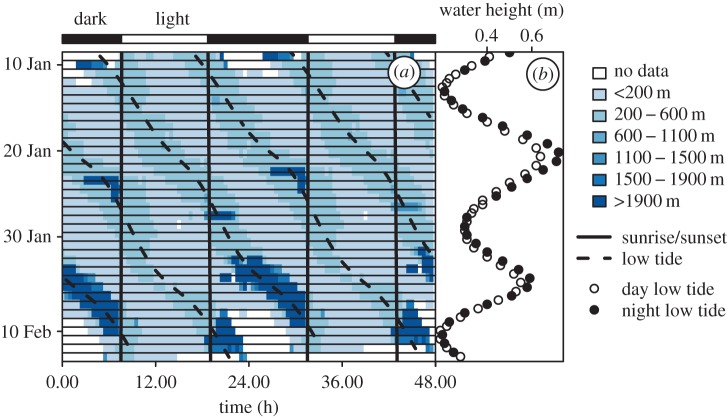
The distance of a radio-tagged red knot to its roost. (*a*) The distance to the main roost (the darker the blue, the farther the knot travelled). Sunrise and sunset are given by the solid vertical lines and the day and night are indicated above the actogram. The low tide times are given by the dashed lines. For actograms see [16]. (*b*) Differences in low-tide water height between day (open circles) and night (filled circles) and during the neap–spring lunar cycle.

We found that the distance of red knots to their roosting site followed the tidal as well as the day–night rhythm (tidal = 88% of individuals, daily = 57%, both rhythms = 52%; *N* = 42 individuals with more than 50 h of observation; median [range] = 19 [2–34] days of observation per individual; for methods see Supplementary Information [16]). At high tide, the birds were generally close to the roost and as the tide retreated, birds moved away from it (figure 2*a*). How far the birds moved was modulated by time of day, but in a bird-specific manner (figure 2*b*). For example, one bird usually roamed between 400 and 600 m from its roost when the low tide occurred during the day (figure 3*a*, light blue), but often went to mudflats further than 1 km from its roost when the low tide occurred at night (figure 3*a*, dark blue). In this particular bird it seems that an approximately 15 day semi-lunar pattern also emerges where the distance travelled at night is greater and is particularly consolidated when the low tide is at its lowest ebb.

The reported tidal rhythms (figure 2*a*) reflect red knots' feeding on molluscs that are only available during low tide. However, why red knots varied so much in how far they travelled during the night and during the day remains unclear. Such daily rhythms (superimposed on the tidal rhythm) can be partly a consequence of the slightly higher tide during the night (figure 3*b*), reducing the maximal extent of the available foraging area. However, why some individuals foraged further from the roost during the night is unclear and unlikely a consequence of dynamics in searching efficiency or food availability. That is, red knots forage by touch rather than by sight [23] and the burying depths of their main prey are not expected to differ between day and night. An alternative explanation for the individual differences may be individual experience with predators. During the day, red knots are predated mainly by large falcons [24,25], and during the night by owls [26–28] . Thus, depending on the local distributions of these two kinds of predators and individual experiences with these predators, the red knot's perceived ‘landscape of fear’ [29], and hence its movement choices, may differ between individuals and between day and night, something worthy of future investigations.

The individuality of red knot tidal movements and hence the investigation of among-individual variation in behavioural rhythms in the wild contrast starkly with laboratory studies where individual subjects, for methodological reasons, are often chosen to be as similar as possible. Although foraging rhythms of red knots appear related to both tidal and daily environmental fluctuations, quantitative studies from different locations are required to validate the generality of these behavioural rhythms, as well as to explore (albeit in a correlative manner) the hypotheses about possible ecological causes of such biorhythms. Also, to demonstrate whether individuals will free-run with circatidal or circadian rhythm or with both of these rhythms, and hence to demonstrate whether these rhythms are truly endogenous, we would need to keep red knots under constant conditions. Such observations will also reveal whether the among-individual differences are endogenous.

### (b) Do tidal shorebirds maintain a tidal incubation rhythm?

In a recent study of 32 species of shorebirds with biparental care, only in 5% of 584 nests did the shorebird pairs display an incubation period length that might have been entrained by the tide [14]. This is surprising, given that half of the studied species live in intertidal habitats away from their breeding grounds [14]. Interestingly, from populations known to forage on intertidal habitats at their breeding grounds (*N* = 10), pairs in only 3 out of 74 nests displayed a period length entrained by the tide. In contrast, incubation rhythms with periods that do not follow the 24 h light–dark cycle were more common and the deviations from 24 h increased in shorebirds breeding at high latitudes.

Although these findings support the existence of a latitudinal cline in incubation rhythms, a substantial number of rhythms defied the 24 h day even at low and mid latitudes. These results might reflect an underestimation of tidal and circadian patterns in incubating shorebirds because the method used depicted only the dominant period of the incubation rhythm, yet other less-dominant periodicities were rare [14]. Importantly, the study suggests that other factors (such as risk of predation and synchronization of the clock between the two parents) might be much more important than any geophysically imposed variable, hence the extremely variable and generally non-daily/tidal rhythmicity in incubation [14].

In summary, these findings suggest that tidal life-history seems to play, at best, a negligible role in determining incubation rhythms, even in shorebirds that forage with the tide during breeding. They corroborate the observations on pre-incubation activities of shorebirds on their Arctic breeding grounds; birds were active around the clock without significant tidal periodicity [30]. Chronobiologists might ask whether these variable cycles of incubation mask an otherwise endogenous circatidal rhythm. Unfortunately, to study any such tidal cycle, birds would have to be removed from the entraining stimuli, conspecifics and any potential predators and placed in free-running constant conditions for several days, something that is impractical during breeding.

## 3. Molecular studies of tidal rhythms

The work described above suggests that tidal and circadian rhythms in foraging shorebirds reflect adjustments to the complex temporal environment in which they live. However, other factors beyond circadian day–night or tidal rhythms, such as predation or behaviour of conspecifics (which themselves may have clock-like features), may outweigh the entrainment of behaviour imposed by these geophysical variables [14]. Still, circadian rhythms are identified in nearly all higher organisms and, for example, migratory birds use the clock for navigation and to compensate for the movement of the sun [31]. Consequently, given the ubiquity of biological rhythmicity, considerable effort has been expended over five decades to identify the genetic and molecular bases for these behavioural rhythms. The discovery of the molecular basis of the circadian clock was a defining moment in the study of gene regulation of complex phenotypes [32].

**Figure 4.**
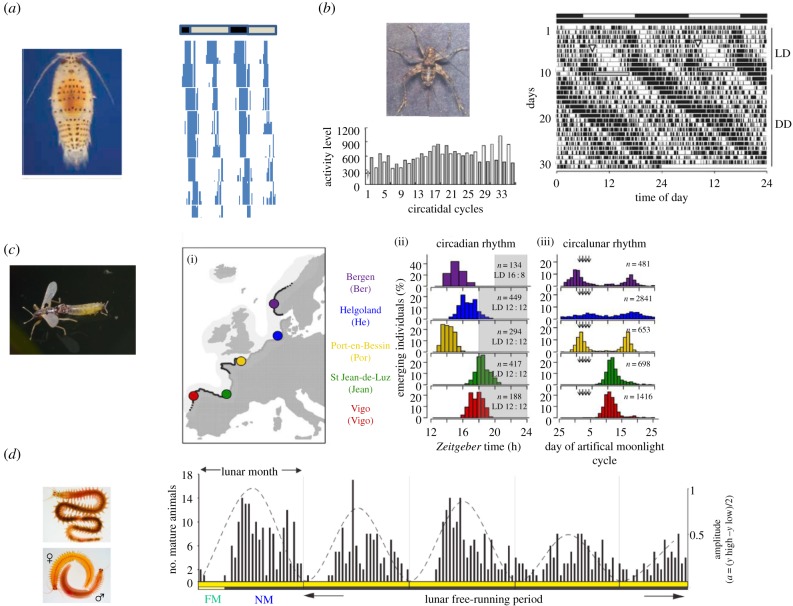
Examples of lunar-related rhythms of invertebrates. (*a*) *Eurydice* adult with chromatophores (black dots on the dorsal surface of cuticle) and swimming activity of a single individual over 9 days in constant darkness). The animal was taken during a spring tide from Bangor, Wales, UK and placed immediately in constant darkness (DD). The approximate natural light (grey)/dark (black) cycle on the day the animal was harvested is shown as a bar above the actogram and each day's activity is double plotted on a horizontal 48 h scale so that so that each row represents two consecutive days. Note that the movement to the right on every successive day reveals a tidal period longer than 12 h and the night-time activity is greater than that of the daytime (diurnal inequality). Adapted from [30]. (*b*) Mangrove cricket and an actogram for single individual placed in 12 L: 12 D for 8 days then allowed to free run in DD, during which there is more intense activity in the dark phase compared with the light phase (see the histogram) which drifts towards the right reflecting the predominantly 24.8–25.5 h rhythm which is about twice the tidal period of approximately 12.4 h. The histogram shows the night-time burst of activity (filled columns) being greater than the daytime burst (unfilled columns) for a few cycles but as this is modulated by the circadian clock, it drifts out of phase with the tidal cycle; so after many cycles, the daytime tidal episode is greater than the night-time (adapted from [33]). The cricket image is taken from http://mangrove.nus.edu.sg/guidebooks/text/2010.htm. (*c*) Midge *C. marinus* and five natural populations (i) with different phases of emergence (ii) and semi-lunar or lunar frequency during day of emergence (iii). Image is taken from https://www.flickr.com/photos/davidh-j/6270311922 and figure was adapted from [34]. (*d*) Premature adult, and adult male and female *Platynereis dumerilii.* Lunar maturation cycle of single individual over several months. FM, full moon simulated by dim light. NM, new moon. Lunar month in days plotted as horizontal yellow bar. Adapted from [35].

Despite insects and crustaceans having long been studied for lunar-related rhythms at the behavioural level [6], we have been missing a genetically tractable model species from intertidal habitats. Here, we introduce four organisms (figure 4) where molecular interventions were recently used to illuminate the molecular bases of lunar-related rhythms. Specifically, we highlight the finding of tidal activity rhythms in the marine isopod *Eurydice pulchra* and the mangrove cricket, *Apteronemobius asahinai*, semi-lunar emergence rhythms of the marine midge, *Clunio marinus*, and the lunar reproductive cycles of the bristle worm *Platynereis dumerilii*.

### (a) Circadian and circatidal rhythms in a marine isopod and a mangrove cricket

*Eurydice pulchra* is a marine isopod that lives in the intertidal zone around northern European coasts (figure 4*a*). As the tide comes in, *Eurydice* swims out of its sandy burrow and forages. As the tide goes out, *Eurydice* buries itself back into the sand so it is not dragged out to sea [33,36]. In constant darkness, *Eurydice* exhibits an endogenous circatidal swimming rhythm of 12.4 h (figure 4*a*) which can be reset by vibration stimuli, and is temperature compensated, thereby showing all the hallmarks of a true clock [36]. Interestingly the swimming pattern usually shows the diurnal inequality phenomenon at temperate latitudes (figure 1*b*), so nocturnal high-tide swimming is considerable greater than daytime swimming (figure 4*a*). This modulation in swimming is regulated by the circadian clock because under bright light it is disrupted, whereas the tidal 12.4 h swimming period is unaffected, suggesting an independence of circadian and tidal oscillators [36].

Moreover, *Eurydice* is called the ‘speckled sea louse’ because it carries pigmented spots, chromatophores that expand during the day and contract at night (figure 4*a*) [33,36]. This 24 h cycle is likely regulated by a circadian clock because the 24 h cycle persists under constant darkness, can be reset by light and is disrupted by constant bright light [33,36]). Indeed, knockdown of *Eurydice*'s *period* gene, whose *Drosophila* orthologue plays a central role in the molecular clock machinery of *Drosophila melanogaster*, has a similar effect to constant light, with circadian cycles in chromatophore dispersion and in *Eurydice timeless* mRNA disrupted. Yet the very same canonical clock gene misregulation has little effect on the circatidal swimming periodicity of 12.4 h [36].

Although these results invoke separate circatidal and circadian oscillators, pharmacological inhibitors of *Eurydice*’s casein kinase 1ɛ (CK1ɛ), which phosphorylates PER protein in *D. melanogaster* and hence could also inhibit similar post-translational modification of *Eurydice*'s PER protein, lengthened both tidal swimming and the circadian chromatophore cycle [36]. This might suggest that the two oscillators share a common pathway. However CK1ɛ has many targets, so the inhibitor might render CK1ɛ less able to phosphorylate a tidally relevant protein that we have yet to identify. It is unlikely that any effect of the inhibitor on *Eurydice*’s PER protein phosphorylation is mediating tidal lengthening because direct disruption of *Eurydice*’s *period* gene mRNA through RNA interference had no effect on this phenotype [36].

The circadian day–night modulation of the tidal swimming rhythms in *Eurydice* is also observed in the locomotor activity of the mangrove cricket [37] (figure 4*b*). However, the periodicity of the cricket's locomotor activity pattern is circatidal and approximately 12.4 h. Elegant genetic studies have used RNAi-mediated knockdown of the canonical clock genes in this species, *period* and *Clock* (in insects and mammals CLOCK protein is one of a pair of molecules that activate *period* and *timeless* gene transcription). The knock-down left 12.4 h tidal rhythms intact, but disrupted the circadian modulation of alternate bouts of locomotor activity [38,39]. As in *Eurydice*, these gene knockdowns suggest that the two molecular oscillators underlying circadian and tidal rhythms are largely independent of each other. Moreover, surgical ablation of the optic lobes (likely location of the circadian oscillator) disrupted the circadian locomotor pattern, but as with the gene knockdown, the tidal rhythm remained intact [34]). Consequently, molecular mechanisms of the two oscillators not only may be independent, but also may reside in different groups of neurons.

### (b) Circadian and semi-lunar emergence of the marine midge

Perhaps the best-known example of a moon-related phenotype in insects is the semi-lunar emergence rhythms in the marine midge, *C. marinus* (figure 4*c*)*,* first studied by Neumann and collaborators 50 years ago (e.g. [40]). During full and new moon, millions of males and females of the midge emerge from the sea as low tide exposes the habitats where they have developed from eggs to pupae (figure 4*c*). These adults mate and live for a few hours, so it is critical that they emerge synchronously during those few hours of low tide. The timing of the lowest tide can be predicted from the lunar calendar, but these critical few hours during the day vary from location to location [40]). Thus, the emergence of the marine midge has to rely on two clocks, one circa-semi-lunar or circalunar, and the other circadian.

A recent and spectacular molecular genetic study used populations of midges living in different European locations (figure 4*c*), in combination with the fully referenced draft genome of the midge generated *de novo* [7], to identify the genetic bases of semi-lunar or lunar and circadian rhythms. First, the local circadian adaptations mapped to the gene encoding calcium/calmodulin-dependent kinase II.1 (*CaMKII*) [7]. Importantly, mutations in the homologous gene can disrupt circadian timing in the mouse [41] and *D. melanogaster* [35,42]. Secondly and more importantly for lunar-related phenotypes, the genetic mapping experiment localized a chromosomal region responsible for the population differences in semi-lunar versus lunar emergence timing [7]. Lack of canonical clock circadian genes mapping to this region implies that a novel timing gene (or genes) contributes to the lunar phenotype.

### (c) Circadian activity and lunar reproductive cycles of the bristle worm

Finally, the bristle worm *P. dumerilii* (figure 4*d*) spawns in a monthly rhythm, in which the number of worms that are sexually mature peaks around the time of new moon and troughs at full moon (figure 4*d*) [43,44]. This monthly rhythm appears to be driven by exposure to moonlight during full moon because the monthly cycle of reproductive maturity can be entrained in the laboratory by nocturnal dim light lasting for eight consecutive nights during the month (figure 4*d*). Also, the monthly maturity rhythm will free-run for several months under constant darkness, but not under constant light or in constant darkness without previous moonlight exposure, suggesting a true circalunar cycle [43]. In addition, the worms show circadian locomotor rhythms particularly in light–dark cycles. The strength of this rhythm is modulated by the phases of the moon, suggesting a crosstalk between the two oscillators [43].

When the worms were treated with the same CK1ɛ/δ kinase inhibitor used in *Eurydice*, circadian locomotor behaviour and circadian gene expression of canonical clock genes were severely disrupted, but the circalunar maturity rhythm was essentially unaffected. The authors' conclusions resonated with those from *Eurydice* and the mangrove cricket, in that the circadian oscillators appeared to be molecularly independent from the circalunar clocks [43]. The only possible inconsistency between the discussed studies concerns tidal and lunar periodicity. The CK1ɛ inhibitor influenced the tidal periodicity in *Eurydice*, but not the lunar cycle in bristle worm. Likely, there are important differences in the mechanisms that generate 12.4 h tidal and 29 day lunar rhythms even though they are clearly geophysically and astronomically related. However, the maturity rhythm of the bristle worm was monitored only for two months after the inhibition. Thus, a period difference between the inhibited and control animals might have gone undetected. It would require several more months of expensive drug exposure and several cycles of monitoring of the maturity rhythm to state definitively that there was no effect on the period of the free-running maturation cycle.

The above examples used molecular manipulations *in vivo* allied to the analysis of behavioural and molecular phenotypes in non-model invertebrates. Such analyses are much more difficult to perform compared with model organisms like *D. melanogaster* or the mouse but they have led to an understanding of what does NOT constitute the tidal oscillator. From three independent studies in *Eurydice,* mangrove crickets and the bristle worm, the consensus of opinion suggests that lunar-related rhythms may not be generated by the canonical circadian clock genes. Some caution should still be reserved in accepting this conclusion, particularly concerning the CK1ε inhibitor, which dramatically affects the period of *Eurydice*’s tidal swimming. In addition, if the tidal oscillator in the mangrove cricket is more robust than the circadian oscillator that modulates its tidal locomotor episodes, then RNAi-mediated knockdown may not knock-down *period* or *Clock* genes far enough to affect the tidal oscillator. Unfortunately, both organisms are difficult to rear in the laboratory so the use of gene editing tools to create null-mutants is unlikely in the near future.

## 4. General conclusion and outlook

We have documented the crosstalk between the tidal and circadian rhythms in the distance that a red knot moved from its roost during foraging (figure 3). This is reminiscent of the circadian modulation of tidal behaviour observed in both *Eurydice* and the mangrove cricket. Thus, we suspect that in all these organisms the brain centres dedicated to expressing tidal and circadian phenotypes will be anatomically connected and, therefore, signalling reciprocally to each other.

The next challenge is to find which genes encode tidal/lunar time in the above-described invertebrates. Once invertebrate lunar/tidal genes are identified, homology should allow the isolation of similar genes in vertebrates like red knots. We might predict that the tidal genes that generate the approximately 12.4 h behavioural cycles might also encode cycling mRNAs by analogy with their circadian counterparts. Might these (as yet unidentified) putatively 12 h tidally cycling mRNAs show among-individual fluctuations to account for the variation in tidal rhythms observed in red knots? Could these mRNAs still be cycling in the biparental incubating species but their output is suppressed? Would any future identification of a tidally cycling mRNA in a tidal vertebrate suggest a co-option of a previously 12 h cycling mRNA in a terrestrial circadian species [45,46] that was re-used to generate tidal phenotypes when the species moved to an intertidal environment?

Whatever the identity of these tidal or lunar genes, the conservation of circadian genes in invertebrates and vertebrates might suggest that the same will be true also for tidal and lunar genes [47]. Tidal genes will initially be identified in invertebrates, but homology with vertebrate genes will be expected to open up interesting possibilities for mechanistic studies of the clock in intertidal birds. For example, using *in situ* hybridization will identify the brain regions that have tidally cycling molecules and comparing these regions with those areas that show circadian cycling molecules will detect both oscillators.

In addition, we must not forget the obvious, that behavioural ecology scenarios are far more complex than those we play out in the confines of the laboratory. As we have learned with shorebirds, individuals vary in their foraging rhythms, and behavioural rhythms during incubation are very loosely coupled to the major environmental cycles [14]. Consequently, the modulation of molecular rhythms by other selection pressures will provide a novel background against which to study biological rhythmicity within an ecologically realistic framework. Indeed, when rodents or flies are placed in semi-natural environments and their circadian rhythms monitored, quite startling results can be observed that could not have been predicted from laboratory studies and which question some of the assumptions made about the adaptive value of the circadian clock [48–50, but see also 51]. As with the incubation study of biparental shorebirds [14], when realistic scenarios are used to study biological rhythms, the results do not meet expectations. We, therefore, encourage behavioural ecologists and chronobiologists to seek collaborations, particularly as the long-term spatial and temporal monitoring of individuals in the field becomes feasible [52] and the new post-genomic age allows molecular study of organisms other than laboratory flies or mice. We anticipate that a fertile hybrid area of research will evolve, perhaps slowly at first, but with a real potential to significantly illuminate our understanding of the functional and adaptive roles of biological rhythms.

## Data accessibility

Information on (methods and actograms) data and results for the red knot foraging and additional incubation results are freely available at https://osf.io/xby9t/ [16]. Data for the shorebird incubation results were retrieved from Open Science Framework, https://osf.io/wxufm/, which also contains further supporting information from the original study [14].

## References

[1] Alerstam T 1990 Bird migration. Cambridge, UK: Cambridge University Press.

[2] Hut RA, Paolucci S, Dor R, Kyriacou CP, Daan S 2013 Latitudinal clines: an evolutionary view on biological rhythms. Proc. R. Soc. B 280, 20130433 (10.1098/rspb.2013.0433) PMC3712436 23825204

[3] de la Iglesia HO, Johnson CH 2013 Biological clocks: riding the tides. Curr. Biol. 23, R921–R923. (10.1016/j.cub.2013.09.006) 24156810 PMC4307598

[4] Dunlap J 2004 Chronobiology. Sunderland, MA: Sinauer Associates.

[5] Naylor E 2010 Chronobiology of marine organisms. Cambridge, UK: Cambridge University Press.

[6] Palmer JD 2000 The clocks controlling the tide-associated rhythms of intertidal animals. Bioessays 22, 32–37. (10.1002/(SICI)1521-1878(200001)22:1%3C32::AID-BIES7%3E3.0.CO;2-U) 10649288

[7] Kaiser TS et al. 2016 The genomic basis of circadian and circalunar timing adaptations in a midge. Nature 540, 69–73. (10.1038/nature20151) 27871090 PMC5133387

[8] del Hoyo J, Elliott A, Sargatal J 1996 Handbook of the birds of the world. Barcelona: Lynx Edicions.

[9] van de Kam J, Ens B, Piersma T, Zwart L 2004 Shorebirds: an illustrated behavioural ecology. Zeist, The Netherlands: KNNV Publishing.

[10] Ens BJ, Underhill LG 2014 Synthesis of oystercatcher conservation assessments: general lessons and recommendations. Int. Wader Stud. 20, 5–22.

[11] Helm B, Gwinner E, Koolhaas A, Battley P, Schwalb I, Dekinga A, Piersma T 2012 Avian migration: temporal multitasking and a case study of melatonin cycles in waders. In *The neurobiology of circadian timing* (eds A Kalsbeek, M Merrow, T Roenneberg, RG Foster), pp. 457–479. Amsterdam: Elsevier. 10.1016/B978-0-444-59427-3.00026-5 22877681

[12] Daan S, Koene P 1981 On the timing of foraging flights by oystercatchers, *Haematopus ostralegus*, on tidal mudflats. Neth. J. Sea Res. 15, 1–22. (10.1016/0077-7579(81)90002-8)

[13] Hötker H 1995 [Activity rhythms of shelducks (*Tadorna tadorna*) and waders (Charadrii) on the North Sea coast]. J. Ornithol. 136, 105–126 (in German with English abstract) (10.1007/BF01651234)

[14] Bulla M et al. 2016 Defying the 24-h day: unexpected diversity in socially synchronized rhythms of shorebirds Nature 540, 109–113. (10.1038/nature20563) 27880762

[15] Foster RG, Wulff K 2005 The rhythm of rest and excess. Nat. Rev. Neurosci. 6, 407–414. (10.1038/nrn1670) 15861183

[16] Bulla M, Oudman T, Bijleveld AI 2017 Supporting information for ‘Marine biorhythms: bridging chronobiology and ecology’ Open Sci. Framework. (10.17605/OSF.IO/XBY9T) PMC5647280 28993497

[17] Zwarts L, Blomert A, Hupkes R 1990 Increase of feeding time in waders preparing for spring migration from the Banc d'Arguin, Mauritania. Ardea 78, 237–256.

[18] Bulla M et al. 2016 Supplementary data 3 – study sites: location, population wing length, monitoring method, tide. Version 11. figshare (10.6084/m9.figshare.1536260.v11)

[19] Piersma T 2007 Using the power of comparison to explain habitat use and migration strategies of shorebirds worldwid*e*. J. Ornithol. 148(Suppl. 1), S45–S59. (10.1007/s10336-007-0240-3)

[20] Buehler DM, Piersma T 2008 Travelling on a budget: predictions and ecological evidence for bottlenecks in the annual cycle of long-distance migrants. Phil. Trans. R. Soc. B 363, 247–266. (10.1098/rstb.2007.2138) 17638692 PMC2606749

[21] van Gils JA, Piersma T, Dekinga A, Battley PF 2006 Modelling phenotypic flexibility: an optimality analysis of gizzard size in red knots (*Calidris canutus*). Ardea 94, 409–420.

[22] Piersma T, Hoekstra R, Dekinga A, Koolhaas A, Wolf P, Battley PF, Wiersma P 1993 Scale and intensity of intertidal habitat use by knots *Calidris canutus* in the western Wadden Sea in relation to food, friends and foes. Neth. J. Sea Res. 31, 331–357. (10.1016/0077-7579(93)90052-T)

[23] Piersma T, van Aelst R, Kurk K, Berkhoudt H, Maas LRM 1998 A new pressure sensory mechanism for prey detection in birds: the use of principles of seabed dynamics? Proc. R. Soc. Lond. B 265, 1377–1383. (10.1098/rspb.1998.0445)

[24] Bijlsma RG 1990 Predation by large falcons on wintering waders on the Banc d'Arguin, Mauritania. Ardea 78, 82.

[25] van den Hout PJ, van Gils JA, Robin F, van der Geest M, Dekinga A, Piersma T 2014 Interference from adults forces young red knots to forage for longer and in dangerous places. Anim. Behav. 88, 137–146. (10.1016/j.anbehav.2013.11.020)

[26] Sitters HP, Gonzalez PM, Piersma T, Baker AJ, Price DJ 2001 Day and night feeding habitat of Red Knots in Patagonia: profitability versus safety? J. Field Ornithol. 72, 86–95. (10.1648/0273-8570-72.1.86)

[27] Rogers DI, Battley PF, Piersma T, Van Gils JA, Rogers KG 2006 High-tide habitat choice: insights from modelling roost selection by shorebirds around a tropical bay. Anim. Behav. 72, 563–575. (10.1016/j.anbehav.2005.10.029)

[28] Piersma T, Gill REJ, de Goeij P, Dekinga A, Shepherd ML, Ruthrauff D, Tibbitts L 2006 Shorebird avoidance of nearshore feeding and roosting areas at night correlates with presence of a nocturnal avian predator. Wader Study Group Bull. 109, 73–76.

[29] Laundré JW, Hernández L, Ripple WJ 2010 The landscape of fear: ecological implications of being afraid. Open Ecol. J. 3, 1–7. (10.2174/1874213001003030001)

[30] Steiger SS, Valcu M, Spoelstra K, Helm B, Wikelski M, Kempenaers B 2013 When the sun never sets: diverse activity rhythms under continuous daylight in free-living arctic-breeding birds. Proc. R. Soc. B 280, 20131016 (10.1098/rspb.2013.1016) PMC3712422 23782884

[31] Beason RC, Wiltschko W 2015 Cues indicating location in pigeon navigatio*n*. J. Comp. Physiol. A Neuroethol Sens. Neural Behav. Physiol. 201, 961–967. (10.1007/s00359-015-1027-2) 26149606

[32] Ozkaya O, Rosato E 2012 The circadian clock of the fly: a neurogenetics journey through tim*e*. Adv. Genet. 77, 79–123. (10.1016/B978-0-12-387687-4.00004-0) 22902127

[33] Wilcockson D, Zhang L 2008 Circatidal clock*s*. Curr. Biol. 18, R753–R755. (10.1016/j.cub.2008.06.041) 18786379

[34] Takekata H, Numata H, Shiga S 2014 The circatidal rhythm persists without the optic lobe in the mangrove cricket *Apteronemobius asahinai*. J. Biol. Rhythms 29, 28–37. (10.1177/0748730413516309) 24492880

[35] Weber F, Hung HC, Maurer C, Kay SA 2006 Second messenger and Ras/MAPK signalling pathways regulate CLOCK/CYCLE-dependent transcription. J. Neurochem. 98, 248–257. (10.1111/j.1471-4159.2006.03865.x) 16805811

[36] Zhang L, Hastings MH, Green EW, Tauber E, Sladek M, Webster SG, Kyriacou CP, Wilcockson DC 2013 Dissociation of circadian and circatidal timekeeping in the marine crustacean *Eurydice pulchra*. Curr. Biol. 23, 1863–1873. (10.1016/j.cub.2013.08.038) 24076244 PMC3793863

[37] Satoh A, Yoshioka E, Numata H 2008 Circatidal activity rhythm in the mangrove cricket *Apteronemobius asahinai*. Biol. Lett. 4, 233–236. (10.1098/rsbl.2008.0036) 18302994 PMC2610048

[38] Takekata H, Matsuura Y, Goto SG, Satoh A, Numata H 2012 RNAi of the circadian clock gene period disrupts the circadian rhythm but not the circatidal rhythm in the mangrove cricke*t*. Biol. Lett. 8, 488–491. (10.1098/rsbl.2012.0079) 22399786 PMC3391462

[39] Takekata H, Numata H, Shiga S, Goto SG 2014 Silencing the circadian clock gene *Clock* using RNAi reveals dissociation of the circatidal clock from the circadian clock in the mangrove cricke*t*. J. Insect Physiol. 68, 16–22. (10.1016/j.jinsphys.2014.06.012) 24995838

[40] Neumann D, Heimbach F 1984 Time cues for semilunar reproduction rhythms in European populations of *Clunio marinus*. II The influence of tidal temperature cycles. Biol. Bull. 166, 509–524. (10.2307/1541158)

[41] Kon N et al. 2014 CaMKII is essential for the cellular clock and coupling between morning and evening behavioral rhythm*s*. Genes Dev. 28, 1101–1110. (10.1101/gad.237511.114) 24831701 PMC4035538

[42] Harrisingh MC, Wu Y, Lnenicka GA, Nitabach MN 2007 Intracellular Ca2^+^ regulates free-running circadian clock oscillation *in vivo*. J. Neurosci. 27, 12 489–12 499. (10.1523/JNEUROSCI.3680-07.2007) PMC6673328 18003827

[43] Zantke J, Ishikawa-Fujiwara T, Arboleda E, Lohs C, Schipany K, Hallay N, Straw AD, Todo T, Tessmar-Raible K 2013 Circadian and circalunar clock interactions in a marine annelid. Cell. Rep. 5, 99–113. (10.1016/j.celrep.2013.08.031) 24075994 PMC3913041

[44] Raible F, Tessmar-Raible K 2014 Platynereis dumerilii. Curr. Biol. 24, R676–R677. (10.1016/j.cub.2014.06.032) 25093553

[45] Akhtar RA, Reddy AB, Maywood ES, Clayton JD, King VM, Smith AG, Gant TW, Hastings MH, Kyriacou CP 2002 Circadian cycling of the mouse liver transcriptome, as revealed by cDNA microarray, is driven by the suprachiasmatic nucleus. Curr. Biol. 12, 540–550. (10.1016/S0960-9822(02)00759-5) 11937022

[46] Hughes ME, DiTacchio L, Hayes KR, Vollmers C, Pulivarthy S, Baggs JE, Panda S, Hogenesch JB 2009 Harmonics of circadian gene transcription in mammal*s*. PLoS Genet. 5, e1000442 (10.1371/journal.pgen.1000442) 19343201 PMC2654964

[47] Clayton JD, Kyriacou CP, Reppert SM 2001 Keeping time with the human genom*e*. Nature 409, 829–831. (10.1038/35057006) 11237000

[48] Gattermann R et al. 2008 Golden hamsters are nocturnal in captivity but diurnal in natur*e*. Biol. Lett. 4, 253–255. (10.1098/rsbl.2008.0066) 18397863 PMC2610053

[49] Daan S et al. 2011 Lab mice in the field: unorthodox daily activity and effects of a dysfunctional circadian clock allel*e*. J. Biol. Rhythms 26, 118–129. (10.1177/0748730410397645) 21454292

[50] Vanin S, Bhutani S, Montelli S, Menegazzi P, Green EW, Pegoraro M, Sandrelli F, Costa R, Kyriacou CP 2012 Unexpected features of *Drosophila* circadian behavioural rhythms under natural condition*s*. Nature 484, 371–375. (10.1038/nature10991) 22495312

[51] Spoelstra K, Wikelski M, Daan S, Loudon AS, Hau M 2016 Natural selection against a circadian clock gene mutation in mice. Proc. Natl Acad. Sci. USA 113, 686–691. (10.1073/pnas.1516442113) 26715747 PMC4725470

[52] Dominoni D, Åkesson S, Klaassen R, Spoelstra K, Bulla M 2017 Methods in field chronobiology. Phil. Trans. R. Soc. B 372, 20160247 (10.1098/rstb.2016.0247) 28993491 PMC5647274

